# The Association

**Published:** 1887-08

**Authors:** 


### THE ASSOCIATION.

Everything seems to promise a pleasant and profitable meeting of the American Dental Association for this year. Some papers of more than usual interest are pledged, and a number of special arrangements have been made which, it it hoped, will prevent some of the friction of previous meetings. There is an excellent opportunity for those who really have the interests of the Association at heart, to prove their loyalty by their attendance and labor this year. It is easily within the limits of possibility to have the most profitable meeting that the society has ever held. But to accomplish this those who attend must drop all selfish and personal ends, and bear in mind that the Association was not organized to further the ambition of any one, but to promote scientific and professional matters. If all will agree to let the constitution and by-laws alone for one year, to allow the Association to elect its own officers without any dictation or wire-pulling, to banish clap-trap and bombast and shun talking for talk's sake, we may have the best meeting ever yet held.

